# A human huntingtin SNP alters post-translational modification and pathogenic proteolysis of the protein causing Huntington disease

**DOI:** 10.1038/s41598-018-25903-w

**Published:** 2018-05-25

**Authors:** D. D. O. Martin, C. Kay, J. A. Collins, Y. T. Nguyen, R. A. Slama, M. R. Hayden

**Affiliations:** 1 Centre for Molecular Medicine and Therapeutics, Department of Medical Genetics, University of British Columbia, Vancouver, BC Canada; 2 Department of Biology, University of Waterloo, Waterloo, Ontario N2L 3G1 Canada

## Abstract

Post-translational modifications (PTMs) are key modulators of protein function. Huntington disease (HD) is a dominantly inherited neurodegenerative disorder caused by an expanded CAG trinucleotide repeat in the huntingtin (*HTT*) gene. A spectrum of PTMs have been shown to modify the normal functions of HTT, including proteolysis, phosphorylation and lipidation, but the full contribution of these PTMs to the molecular pathogenesis of HD remains unclear. In this study, we examine all commonly occurring missense mutations in *HTT* to identify potential human modifiers of HTT PTMs relevant to HD biology. We reveal a SNP that modifies post-translational myristoylation of HTT, resulting in downstream alterations to toxic HTT proteolysis in human cells. This is the first SNP shown to functionally modify a PTM in HD and the first validated genetic modifier of post-translational myristoylation. This SNP is a high-priority candidate modifier of HD phenotypes and may illuminate HD biology in human studies.

## Introduction

Huntington disease (HD) is a debilitating neurodegenerative disease with no treatment to delay progression of the disease. HD is caused by an expanded CAG repeat in the huntingtin gene (*HTT*) that translates into a polyglutamine tract at the N-terminus of the huntingtin protein (HTT)^1^. Wild-type HTT is critical for cell viability^2^ and has been shown to play a role in a variety of pathways including orientation of the mitotic spindle^3^, trafficking of autophagosomes in neurons^4^, and in regulating autophagy^5^. Expanded polyglutamine in HTT leads to loss of function and a toxic gain of function in neurons of the striatum and cortex, causing behavioral changes, movement deficits and ultimately death. Age of disease onset is inversely proportional to the length of the CAG repeat^6^. However, age of onset may be accelerated or delayed by genetic modifiers, including a SNP at the transcription factor binding site of Nf-kB in the *HTT* promoter^7^, and in genes related to DNA repair^8^.

The HTT protein is a large monomeric protein whose function is intricately regulated by post-translational modifications including phosphorylation, acetylation, ubiquitination, proteolysis, and fatty acylation^9^. While some PTMs of HTT have been shown to be protective against toxicity of mutant HTT, such as phosphorylation at S13/16 and S421^10^, others are crucial for HD pathogenesis or increase mutant HTT toxicity. In particular, caspase-mediated proteolysis of HTT at amino acid D586 has been shown to be necessary for the development of disease phenotypes in HD mouse models^11,12^. Consequently, modulating PTMs has become a focus of therapeutic strategies for HD. We sought to identify human SNPs that lead to missense mutations that may alter PTMs in HTT and, consequently, modify progression or pathogenic effects of the disease.

## Results

To identify SNPs that could alter HTT PTMs and potentially modify HTT function, all common missense mutations (≥0.1% minor allele frequency; MAF) within *HTT* were curated from Phase 3 of the 1000 Genomes Project (1 KG) and from the Genome Aggregation Database (gnomAD). Nineteen common missense SNPs with ≥0.1% MAF were found in 1 KG, and 19 common missense SNPs with ≥0.1% MAF were found in gnomAD (Table 1). The top 14 most common missense SNPs in 1 KG and in gnomAD were shared in both data sets, highlighting convergent allele discovery by distinct methodologies.

**Table 1:** Functional SNPs in gnomAD and 1000 Genomes Phase 3.

RSID	Chromosome Position (GRCh37.p13)	Reference	Alternate	Amino acid position (NP_002102.4)	Mutation	Allele Frequency (gnomAD)	Allele Frequency (1000 G)	PROVEAN^27^ PREDICTION	SIFT^28^ PREDICTION	SNAP^29^ PREDICTION
rs362331	3215835	T	C	2311	Y/H	0.43680	0.44129	Neutral	Tolerated	Neutral
rs362272	3234980	G	A	2788	V/I	0.28390	0.21026	Neutral	Tolerated	Neutral
rs363125	3189547	C	A	1722	T/N	0.12750	0.18910	Neutral	Tolerated	Neutral
rs149109767	3230410	AGAG	A	2645	E/–	0.06067	0.02656	Deleterious	NA	—
rs35892913	3148570	G	A	1066	V/I	0.04200	0.02356	Neutral	Tolerated	Neutral
rs363075	3137674	G	A	895	G/R	0.04069	0.02376	Deleterious	Damaging	Effect
rs1143646	3148653	T	G	1093	I/M	0.03962	0.02236	Neutral	Damaging	Neutral
rs3025837	3174845	A	C	1387	N/H	0.01489	0.04153	Neutral	Tolerated	Neutral
rs34315806	3162034	C	T	1262	T/M	0.00837	0.02756	Neutral	Damaging	Neutral
rs151106561	3132038	A	G	627	Q/R	0.00523	0.00280	Neutral	Tolerated	Neutral
rs150003356	3189510	T	C	1710	C/R	0.00281	0.00100	Deleterious	Tolerated	Effect
rs3025843	3156038	A	G	1175	T/A	0.00256	0.00679	Neutral	Tolerated	Neutral
rs118005095	3129240	G	A	553	G/E	0.00242	0.00619	Neutral	Tolerated	Neutral
rs34389685	3133113	G	A	698	G/E	0.00190	0.00200	Deleterious	Damaging	Effect
rs186355914	3109095	C	T	233	A/V	0.00170	0.00020	Neutral	Damaging	Neutral
rs201702168	3221998	A	T	2446	E/D	0.00154	0.00140	Neutral	Tolerated	Neutral
rs375957195	3230428	C	A	2647	D/E	0.00153	0.00319	Neutral	Tolerated	Neutral
rs1065746	3148624	G	C	1084	D/H	0.00135	0.00020	Deleterious	Damaging	Effect
rs190593027	3144528	C	G	996	T/R	0.00110	0.00140	Neutral	Damaging	Neutral
rs201646519	3210567	T	C	2076	S/P	0.00018	0.00100	Neutral	Tolerated	Neutral
rs201760820	3134411	G	A	789	V/M	0.00047	0.00100	Neutral	Tolerated	Neutral

**Figure 1:**
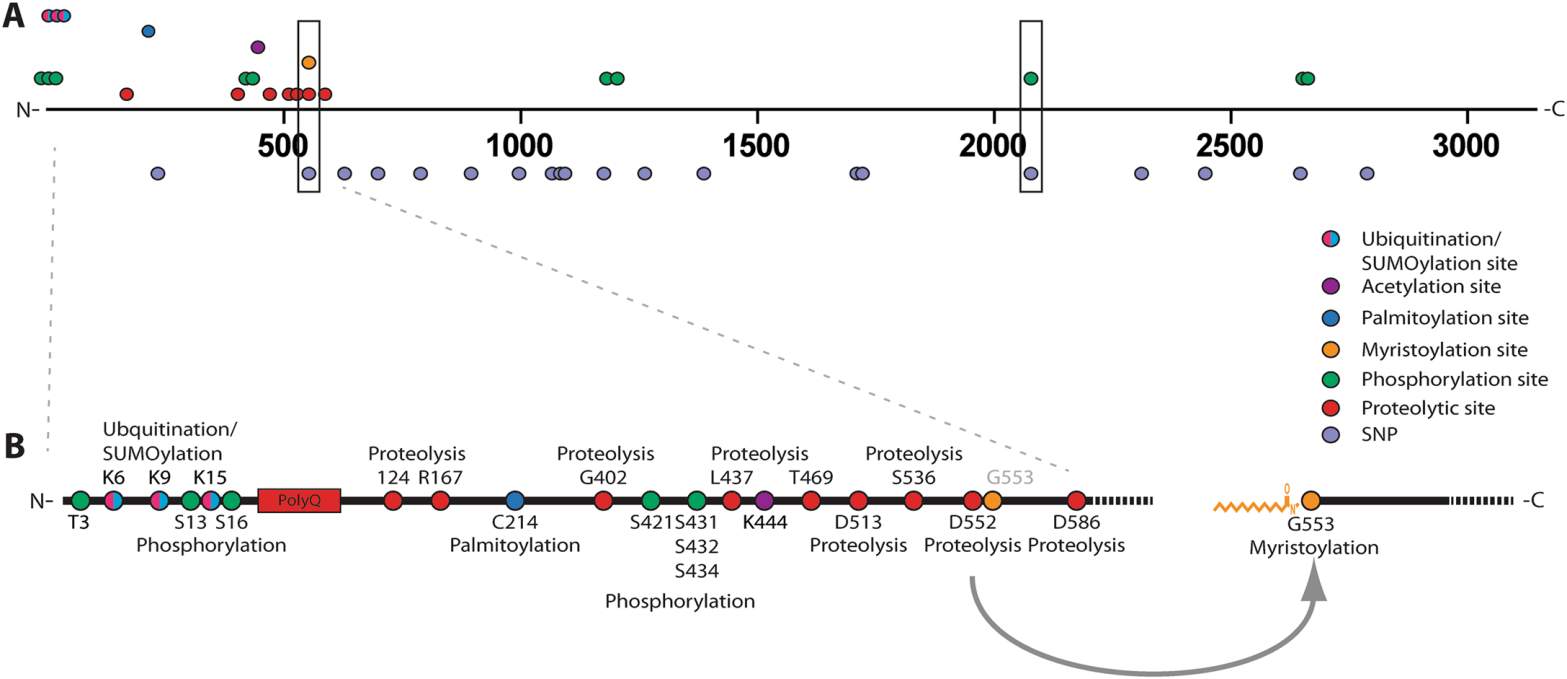
Schematic representing where HTT PTMs and *HTT* missense SNPs intersect. (**A**) Linear map of *HTT* SNPs leading to missense mutations mapped to known HTT PTMs. The two missense mutations that directly intersect with myristoylation at G553 and phosphorylation at S2076, G553E and S2076P, are boxed. (**B**) PTMs within the first 586 amino acids of HTT are highlighted. Proteolytic caspase sites are indicated on the bottom while non-caspase mediated proteolytic sites are displayed on top. G553 is myristoylated following caspase cleavage at D552.

**Figure 2:**
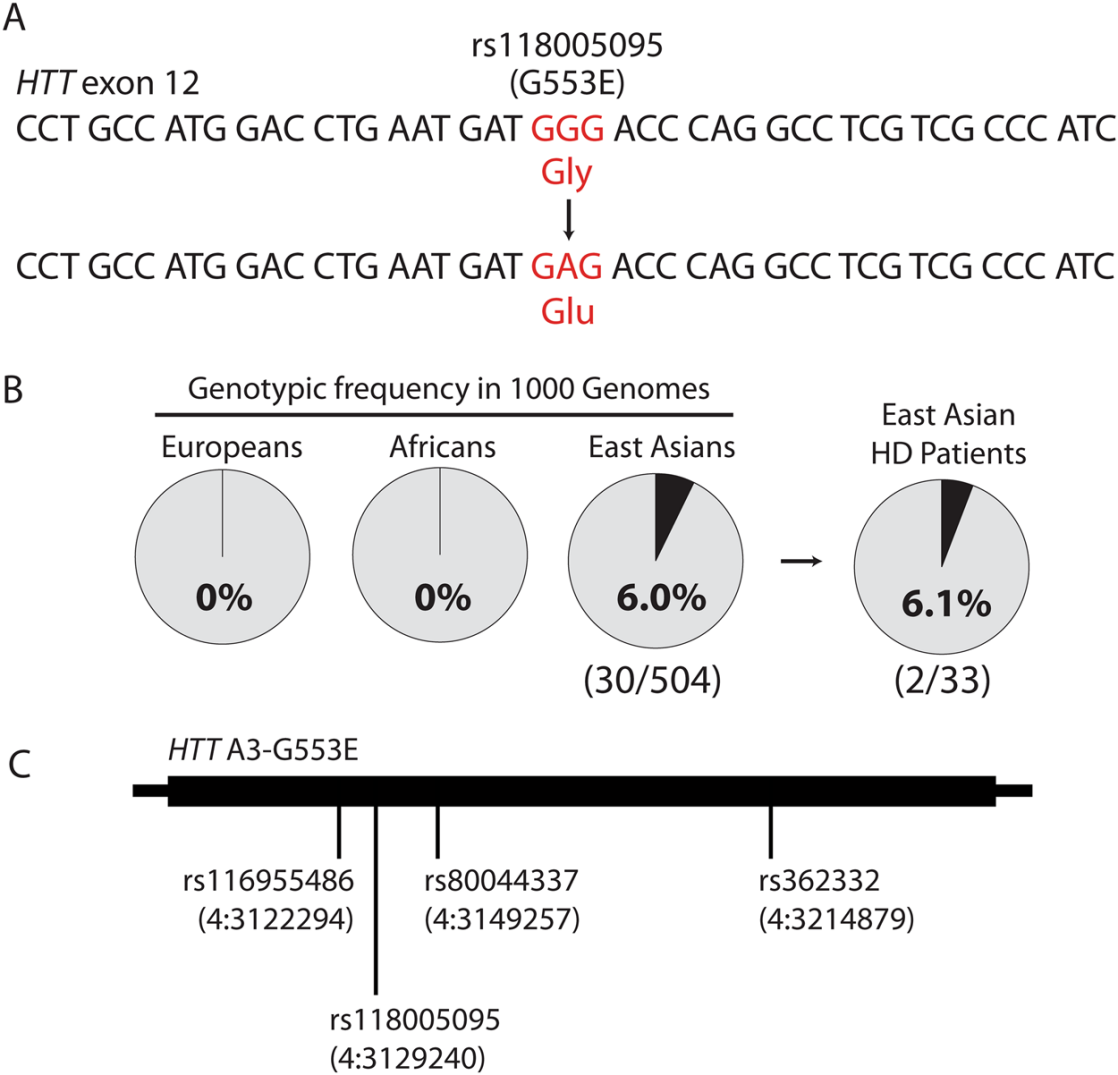
The rs118005095 missense variant is a naturally occurring human SNP that alters the HTT amino acid sequence. (**A**) rs118005095 results in mutation of the HTT 553 glycine residue to glutamic acid. (**B**) rs118005095 occurs exclusively in populations of East Asian ancestry. (**C**) The rs118005095 variant is one of four SNPs defining a gene-spanning *HTT* haplotype in the East Asian population.

A manually curated list of known HTT PTMs was intersected with all common missense SNPs identified from 1 KG and gnomAD (Fig. 1). Among the 21 common missense SNPs identified from 1 KG and gnomAD, two overlapped directly with known HTT PTMs; rs118005095 (G553E) and rs201646519 (S2076P) (Table 1, boxed; Supplemental Table 1). SNP rs201646519 intersects with the predicted phosphorylated residue S2076, resulting in a proline substitution thereby blocking phosphorylation at this site. Myristoylated G553^13,14^ overlapped with the missense SNP rs118005095, which leads to a substitution of glycine to glutamic acid (G553E) and would therefore completely abrogate post-translational myristoylation at this site (Fig. 2A). Myristoylation involves the covalent addition of the 14 carbon fatty acid, myristate, to N-terminal glycines either co-translationally on the nascent polypeptide as it is being translated on the ribosome following the removal of the initiator methionine or post-translationally following proteolysis that exposes an N-terminal glycine^15^. The fatty acid moiety promotes membrane binding of proteins. No other common missense SNPs directly intersected with a known HTT PTM residue. Due to the low frequency of rs201646519, we focused on rs118005095.

The G553E SNP rs118005095 shows pronounced ethnic differences in frequency, being most common in individuals of East Asian ancestry. In 1 KG, rs118005095 is observed on 31 out of 5008 chromosomes from all populations, of which 30 instances occur in subjects of defined East Asian origin (n = 504 subjects) at a genotypic frequency of 6.0% (30/504) and allelic frequency of 3.0% (30/1008) (Fig. 2B). The one remaining 1 KG chromosome with rs118005095 outside East Asian individuals occurs in a Bengali subject from Bangladesh, close to East Asia. In the gnomAD data, rs118005095 occurs in chromosomes from East Asian subjects at an allelic frequency of 2.914% (552/18942). In contrast, rs118005095 is observed in <0.1% of chromosomes from European, African, South Asian, and Latino subjects, reflecting its absence in similar reference populations from 1 KG. Therefore rs118005095 is expected to occur in approximately 6.0% of individuals from the East Asian general population.

We have previously shown that *HTT* is characterized by a haplotype block of low recombination and that SNPs within the gene represent specific haplotypes^16^. *HTT* haplotype analysis in 1 KG reveals that rs118005095 occurs on a specific A3b haplotype variant in the East Asian population, tagged by three additional haplotype-defining SNPs: rs116955486, rs80044337, and rs362332 (Fig. 2C). Of note, previously published haplotypes of the HD mutation in East Asian patients do not include this haplotype, suggesting that this SNP is not found on mutant *HTT* chromosomes in the East Asian population. We thus predicted that rs118005095 may occur in wild-type *HTT* of East Asian HD patients, potentially impacting the normal function of myristoylation in the context of the disease.

We have previously shown that the expanded polyglutamine mutation dramatically reduces myristoylation of HTT at G553^14^. HD patients with the G553E mutation (rs118005095) would therefore be unable to compensate for deficient myristoylation with a wild-type copy bearing the rs118005095 variant. Genotyping of archived DNA from 33 East Asian HD patients in the UBC HD Biobank confirmed the presence of the SNP in two patients (Fig. 2B). As expected, phasing of rs118005095 to the mutation revealed that this missense variant occurred on wild-type *HTT* in these patients. However, no clinical information was available to assess the potential modifying effects of the SNP.

**Figure 3:**
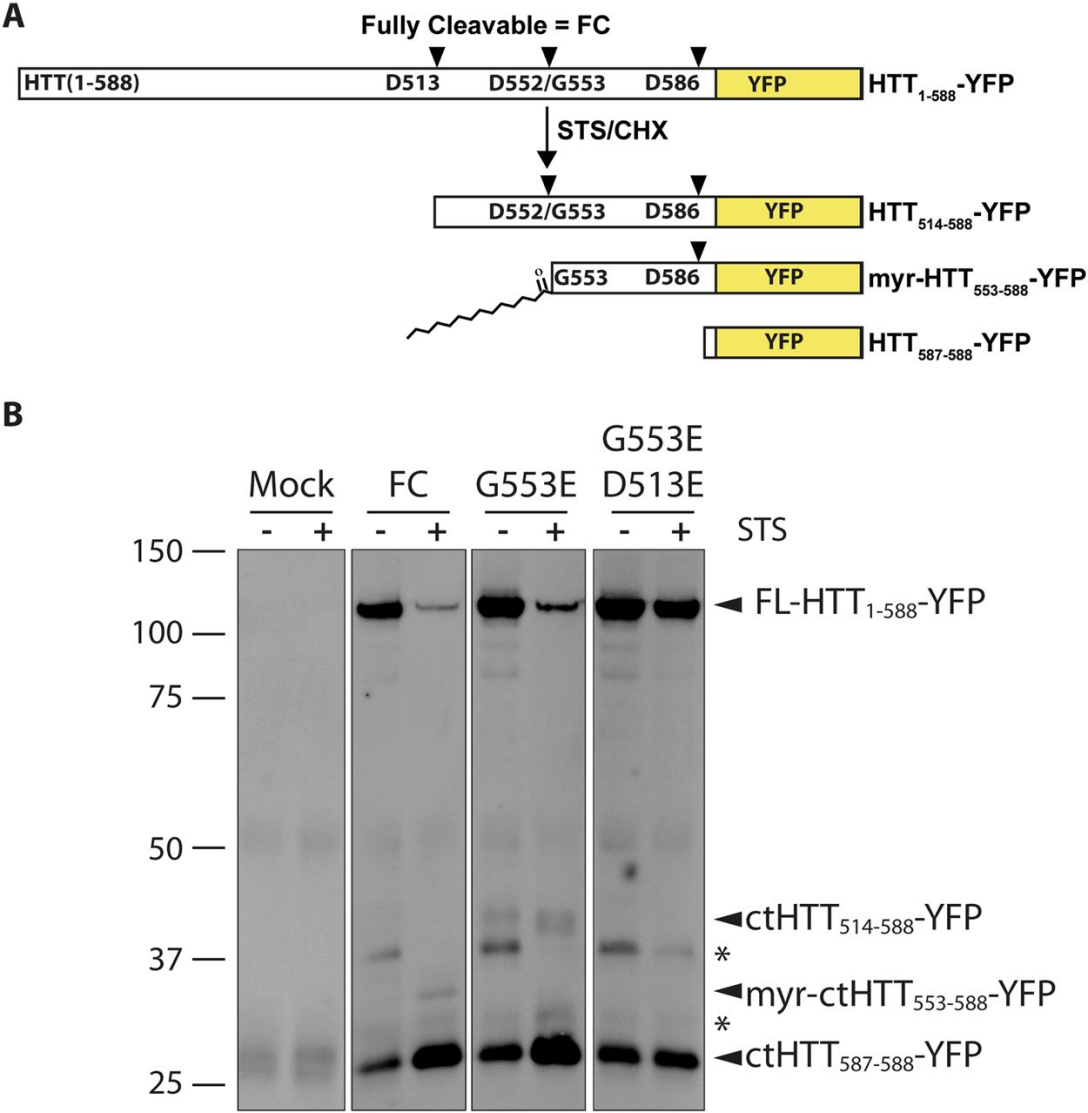
The G553E substitution blocks post-translational myristoylation and alters proteolysis of wtHTT. (**A**) Schematic representation of proteolytic cleavage of WT fully cleavable (FC) HTT_1–588_-YFP. HTT is proteolysed by caspases at D586 and D552 and post-translationally myristoylated at G553. (**B**) HeLa cells were transfected with WT HTT_1–588_-YFP bearing the indicated point mutations. Proteolysis was induced with staurosporine (STS) and cycloheximide. In the presence of the G553E mutation, a higher molecular weight band was detected that was blocked when D513 was mutated to E, confirming the D513 caspase cleavage site. *Denotes unknown or non-specific bands. Image is a composite of lanes from the same Western blot.

To investigate the predicted cellular effects of the rs118005095 (G553E) variant, we generated the mutation in a biologically relevant form of HTT bearing the first 1–588 amino acids appended to YFP (HTT_1–588_-YFP, Fig. 3A), previously shown to be post-translationally myristoylated^14^ and disease-causing in HD mouse models^17^. Myristoylation at G553 was first validated in WT and mHTT_1–588_-YFP using an alkyne-myristate analog that can be covalently linked to biotin-azide via Click chemistry for detection (Supplementary Fig. 1)^18^. Caspase-cleavage at D552 was promoted using staurosporine (STS) and cycloheximide (CHX) in order to increase post-translational myristoylation at G553, as previously described^14^. Post-translational myristoylation was detected in fully-cleavable (FC) HTT (HTT_1–588_-YFP with no point mutations added) even in the absence of STS/CHX, likely due to basal levels of caspase activity (Supplementary Fig. 1). Myristoylation was increased in the presence of STS/CHX. A point mutation at the primary site of caspase-cleavage in HTT at position D586 to glutamate was made in order to more easily measure myristoylation at G553 following caspase cleavage at D552 (D586E, Supplementary Fig. 1). This led to a dramatic increase of post-translational myristoylation at G553.

As predicted, substitution of the essential glycine to alanine or serine (G553A/D586E and G553S/D586E) completely abrogated post-translational myristoylation of HTT at G553 following caspase-induced cleavage. Similarly, blocking caspase cleavage at D552 by substituting the aspartate to glutamate (D552E, Supplementary Fig. 1) completely blocked post-translational myristoylation at G553. Inhibiting caspase-cleavage at D552 appeared to block the generation of both the myristoylated and non-myristoylated bands. Therefore, we determined that myristoylation status at G553 could also be determined by gel mobility. Non-myristoylated C-terminal (ct) HTT_553–588_-YFP migrates at a slightly higher molecular weight than myristoylated-ctHTT_553–588_-YFP (Supplementary Fig. 1). This mobility shift was shown previously in a shorter myristoylated fragment^14^ and mimics other lipidated proteins such as LC3^19^.

**Figure 4:**
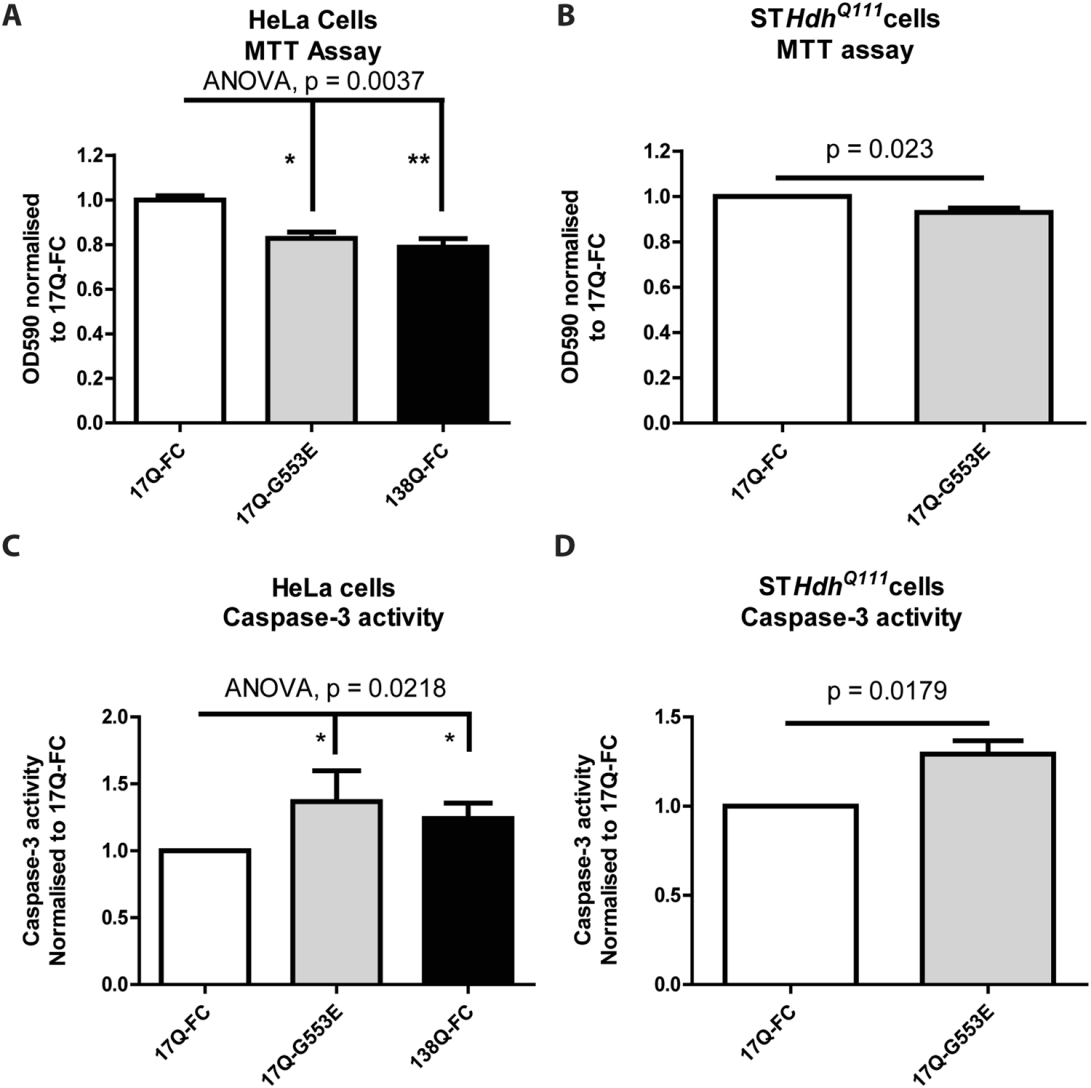
The G553E mutation leads to decreased cell viability and increased caspase-3 activity in HeLa cells and the HD cell model ST*Hdh*^*Q111*^ cells. (**A**,**B**) The MTT assay was used to measure cell viability in cells expressing the indicated constructs. (**A**) HeLa cells (ANOVA, p = 0.0037, h = 11.22, n = 6, 2–3 replicates per n). (**B**) STHdh^Q111^ cells (paired t-test, p = 0.0234, n = 5, 3 replicates per n). (**C**,**D**) Caspase-3 activity was measured in cells expressing the indicated constructs by following cleavage of the fluorescent substrate Ac-DEVD-AFC. Caspase-3 activity is represented as relative fluorescence units normalised to 17Q-FC. (**C**) HeLa (ANOVA, p = 0.0218, h = 7.652, n = 5, 3 replicates per n). (**D**) STHdh^Q111^ cells (paired t-test, p = 0.0179, n = 5, 3 replicates per n). (**A**) and (**C**) Represent Kruskal-Wallis non-parametric ANOVA with Dunn’s Multiple comparison test post hoc analysis. *p < 0.05, **p < 0.01). (**B**) and (**D**) Represent 2-tailed paired Student’s t-test. Error bars represent SEM.

Consequently, the G553E mutation was further characterized in the absence of the myristate analog in WT-HTT_1–588_-YFP (Fig. 3). As expected, substitution of the essential N-terminal glycine at position 553 completely abrogated post-translational myristoylation of the wild-type form (Fig. 3B). Surprisingly, the G553E mutation also inhibited caspase cleavage of HTT at D552, which was associated with a concomitant increase in caspase cleavage of HTT at D513 (Fig. 3B). Cleavage at D586 did not appear to be affected. Of note, blocking myristoylation conventionally by substituting glycine to alanine did not prevent cleavage at D552 (Supplemental Fig. 1). Proteolysis at D513 was recently shown to be toxic in cells^11^. Due to the increased cleavage of HTT at the toxic site D513 in the presence of the G553E mutation, we hypothesized that the G553E variant may induce toxicity of wild-type HTT. Indeed, HeLa cell viability, measured by the MTT assay, was significantly reduced, similar to mutant 138Q HTT alone, in the presence of the G553E mutation compared wild-type HTT (Fig. 4A). Toxicity was also evaluated by measuring cleavage of spectrin (Supplemental Fig. 2), a common marker for cell death, particularly in HD studies^20–22^. Spectrin cleavage was significantly increased (Supplemental Fig. 2) in HeLa cells expressing mHTT, while the G553E mutation had an intermediate effect corresponding to the cell viability MTT assay. Cell viability was also significantly decreased in ST*Hdh*^*Q111*^ cells in the presence of the G553E mutation compared to wild-type FC-HTT (Fig. 4B). These cells are striatal derived cells from transgenic mice in which human exon 1 has been inserted into the mouse gene and represent a cellular model for HD^23^. In agreement with the cell viability MTT assay, we detected a significant increase in caspase-3 activity in HeLa cells expressing the 17Q-HTT-G553E-YFP or mHTT (Fig. 4C), suggesting the intiation of apoptosis. Similarly, the G553E substitution significantly increased caspase-3 activity in ST*Hdh*^*Q111*^ cells (Fig. 4D). Altogether, this suggests that expression of G553E mutation is toxic alone and toxicity is increased in the presence of the disease causing polyQ mutation.

## Discussion

Previously, we have shown that proteolysis at D586 is essential to the pathogenesis of HD in a mouse model^12^. In addition, we identified post-translational myristoylation as a novel PTM in HTT that is decreased in the presence of the expanded polyQ suggesting it is a protective PTM^14^. Now, we have identified the first SNP leading to a missense mutation that alters multiple PTMs in HTT. This is also the first demonstration of a naturally occurring missense mutation that blocks post-translational myristoylation. The G553E mutation was shown to completely abrogate post-translational myristoylation of HTT and induce cellular toxicity of the protein *in cellulo*. Surprisingly, this mutation gives rise to a toxic fragment of wild-type HTT caused by proteolysis at D513.

We have shown the G553E mutation blocks caspase cleavage at D552 leading to increased cleavage of HTT at the toxic caspase site D513. It has recently been shown that proteolysis of either wild-type HTT or mutant HTT at more than one site, particularly caspase-cleavage at D513 and D586, is toxic^11^. This is mediated by a newly appreciated toxicity associated with the C-terminal HTT that is exacerbated by toxic shorter N-terminal fragments^11,24^. It is thought that the N- and C-terminal fragments interact in the presence of single site cleavage, which is considered less toxic. However, increased cleavage at multiple sites leads to decreased interaction following proteolysis at more than one site, particularly caspase cleavage at D513 and D586 and calpain cleavage at R167^11^. This proteolysis is toxic in both wild-type and mutant HTT.

**Figure 5:**
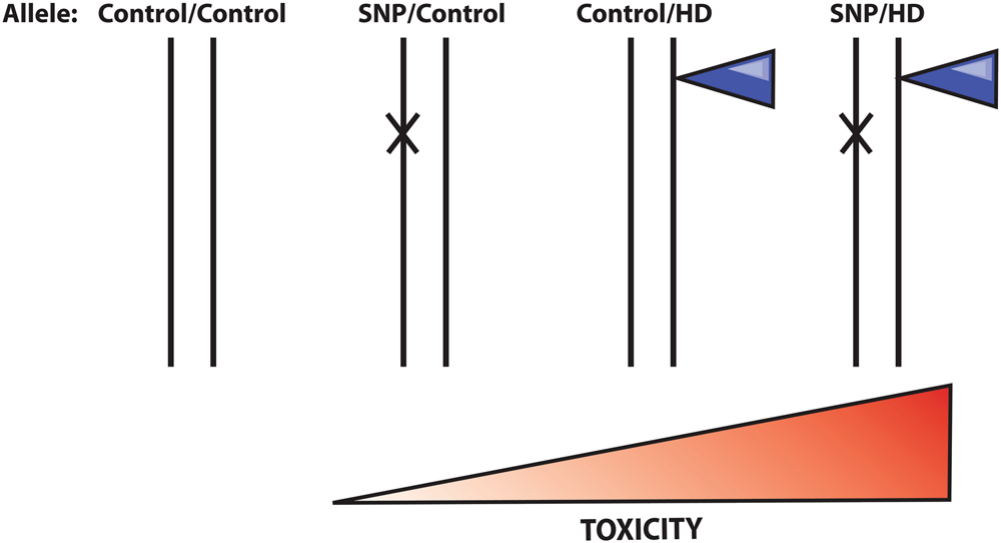
Allelic representation of the compound effect of the G553E mutation with the HD mutation. HD is a monogenic disease caused by a CAG expansion. The SNP encoding the G553E mutation occurs on the control allele causing toxicity. It is predicted that carriers of both mutations with have an earlier onset of disease.

Consequently, increased proteolysis of wild-type HTT at D513 caused by the G553E mutation, in conjunction with cleavage at D586 and loss of protective myristoylation, is predicted to be toxic. Loss of function of the mutant HTT allele would likely exacerbate this toxicity (Fig. 5), which is supported by the increased toxicity when the G553E mutation is expressed in the presence of the HD mutation in ST*Hdh*^*Q111*^ cells (Fig. 4B and D). Remarkably, blocking cleavage at D586 in an HD mouse model has been shown to completely ameliorate the disease phenotype in mice^12^. Therefore, we predict that the G553E mutation may be an important disease modifier and will lead to an earlier onset of disease in HD patients. It will be important to screen East Asian HD patients for the G553E mutation to validate the proteolytic consequences and phenotypic impact of the rs118005095 variant.

## Materials and Methods

### Genotyping and Haplotype Analysis

Haplotype analysis of rs118005095 in 1000 Genomes Phase 3 was performed using custom scripts in the R statistical computing environment applied to a variant call file spanning *HTT* variants (chr4:3034088-3288007) in all 5008 subjects^16^. SNPs in high pairwise linkage disequilibrium (r^2^ > 0.90) with rs118005095 were found in similarly high linkage disequilibrium on a common gene-spanning haplotype in subjects of East Asian ancestry among all populations in 1000 Genomes. Direct genotyping of rs118005095 was performed on banked DNA samples from East Asian HD subjects from the UBC HD Biobank. PCR amplicons containing the G553E variant (rs118005095) were generated using rs118005095 forward (GCGGACTCAGTGGATCTGGC) and rs118005095 reverse (GTCTGAAGGGGTAACAGCTGAATC) primers and Sanger sequenced using one or both primers. All subject DNA samples were collected, stored and examined with informed consent and approval of the University of British Columbia, Children’s and Women’s Health Centre of British Columbia and the Vancouver Coastal Health Research Institute’s Research Ethics Boards, (UBC-C&W H06-70467 and H06-70532, VCHRI V09-0129). The study of both previously collected de-identified archived DNA samples from the HD BioBank at UBC as well as patient samples collected from the Centre for HD at UBC was conducted in accordance with the protocols and procedures required by UBC Clinical Research Ethics Board and their affiliated REBs.

### Cloning

All HTT-YFP point mutations were generated using gBlocks, as described previously^25^, except the D513E mutation was generated by Top Gene Technologies. All point mutations were confirmed by DNA sequencing (Eurofins Genomics).

### Click chemistry and fragment analysis

Post-translational myristoylation of HTT_1–588_-YFP was detected as described previously^14^. Briefly, HeLa cells were transiently transfected using X-tremeGene (Roche) as per the manufacturer’s directions. After 20 h post-transfection, the media was exchanged with DMEM containing charcoal filtered FBS and cells were incubated with alkyne-myristate (Cayman Chemicals) for 30 mins prior to the addition of 1 µM staurosporine (STS) and 5 µg/mL of cycloheximide (CHX) to promote caspase activity and prevent synthesis of new protein, respectively. After 4 h, cells were lysed in modified RIPA buffer. HTT_1–588_-YFP was immunoprecipitated using goat anti-GFP (Eusera) and subjected to Click chemistry using azido-biotin (Invitrogen). Following SDS-PAGE on large 10% gels, post-translational myristoylation was detected by Western blot analysis using streptavidin Alexa680 (Invitrogen) and Licor. YFP was detected using rabbit anti-GFP (Eusera).

### Viability assays

Cell viability was measured using the MTT assay (Sigma) as per the manufacturer’s directions. Briefly, 20 h post-transfection in HeLa cells and 44 h in StHdh(Q111) cells, MTT was added for an additional 4 h. OD readings were normalized to WT fully cleavable HTT. The experiment was performed 5–6 times with 3 technical replicates per experiment for each condition. Alternatively, toxicity of each construct was evaluated by measuring cleavage of spectrin. As above, HeLa cells were transfected with the indicated constructs for 24 h, lysed in RIPA buffer, lysates were separated on 7% SDS-PAGE gels, transferred to PVDF, probed for spectrin (AA6, Enzo Life Sciences) and detected by Licor. Full-length and cleaved spectrin were quantified using Image Studio Lite V.4 and presented as a ratio of cleaved spectrin over total spectrin (n = 4).

Caspase-3 activity was measured as previously described^26^. Briefly, adherent and floating cells were collected 24 h post-transfection and lysed (50 mM Tris pH 8, 150 mM NaCl, 1% Igepal). Lysates were diluted in 2X caspase assay buffer (100 mM HEPES pH 7.4, 200 mM NaCl, 0.2% CHAPS, 2 mM EDTA and 20% glycerol). Fresh DTT (20 mM) and 200 µM Ac-DEVD-AFC (Enzo Biosciences) was added in a 384-well plate. The relative fluorescence units (RFU)/min were acquired by measuring fluorescence every 2–5 min with excitation at 400 nm and emission at 505 nm.

### Data availability

Data described in this study will be available from the corresponding authors upon request.

## Electronic supplementary material

Supplementary Data
