# Supplementary material for: A human huntingtin SNP alters post-translational modification and pathogenic proteolysis of the protein causing Huntington disease
Source: Sci Rep. 2018 May 25;8:8096. doi: 10.1038/s41598-018-25903-w (PMC5970160; doi:10.1038/s41598-018-25903-w)
Supplement: Supplementary file 1 — Supplementary Data [file 41598_2018_25903_MOESM1_ESM.pdf]

Supplementary Material:

A human huntingtin SNP alters post-translational modification and pathogenic proteolysis of the protein causing Huntington disease

Martin DDO[1,2]*, Kay C[1], Collins JA[1], Nguyen YT[1], Slama RA[1], Hayden MR[1]*

[1]Centre for Molecular Medicine and Therapeutics, Department of Medical Genetics, University of British Columbia, Vancouver, BC, Canada

[2]Present address: Department of Biology, University of Waterloo

***Corresponding authors:**

Dr. Dale Martin

dale.martin@uwaterloo.ca

Dr. Michael R. Hayden

mrh@cmmt.ubc.ca

Phone: +1 604 875 3535

Fax: +1 604 875 3819

Supplemental Table 1 – List of established PTMs[1,2] and novel missense mutations. Amino acid positions refer to NP_002102.4.

PTM Position	Amino acid	PTM	Missense mutation position	Original AA	Effect
3	T	phosphorylation			
6	K	Sumoylation			
6	K	ubiquitination			
9	K	Sumoylation			
9	K	ubiquitination			
13	S	phosphorylation			
15	K	Sumoylation			
15	K	ubiquitination			
16	S	phosphorylation			
81-129		proteolysis			
104-114		proteolysis			
124		proteolysis			
167	R	proteolysis			
205-214		proteolysis			
214	C	palmitoylation			
			233	A	V
402	G	proteolysis			
421	S	phosphorylation			
431	S	phosphorylation			
432	S	phosphorylation			
434	S	phosphorylation			
437	L	proteolysis			
444	K	acetylation			
469	T	proteolysis			
513	D	proteolysis			
536	S	proteolysis			
552	D	proteolysis			
553	G	myristoylation	553	G	E
586	D	proteolysis			
			627	Q	R
			698	G	E
			789	V	M
			895	G	E
			996	Y	R
			1066	V	I
			1084	D	H
			1093	I	M
			1175	T	A
1181	S	phosphorylation			
1201	S	phosphorylation			
			1262	T	M
			1387	N	H
			1710	C	R
			1722	T	N
2076	S	phosphorylation	2076	S	P
			2311	Y	H
			2446	E	D
			2645	D	del
			2647	D	E
2653	S	phosphorylation			
2657	S	phosphorylation			
			2788	V	I

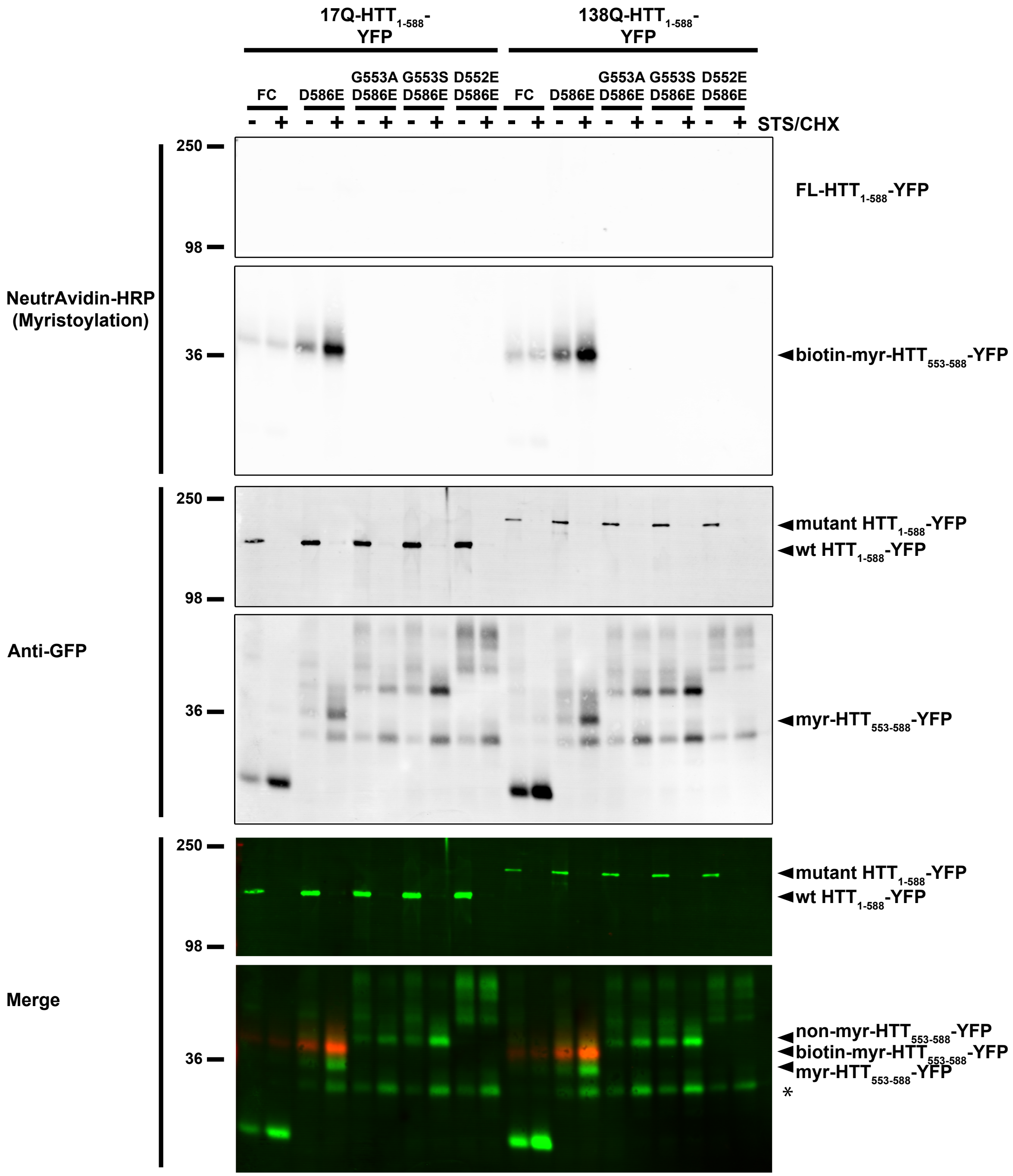

Supplementary Figure 1 – Orthogonal detection of post-translational myristoylation in $HTT_{1\text{-}588}$-YFP. $HTT_{1\text{-}588}$-YFP was immunoprecipitated from HeLa cells incubated with alkyne-myristate. The myristate analog was covalently linked to azido-biotin through Click chemistry for detection. Biotinylated myristoylated cleaved HTT (biotin-myr-$HTT_{553\text{-}588}$-YFP) migrated at an intermediate molecular weight between unlabeled myristoylated cleaved HTT (myr-$HTT_{553\text{-}588}$-YFP) and non-myristoylated cleaved HTT (non-myr-$HTT_{553\text{-}588}$-YFP). Both biotin-myr-$HTT_{553\text{-}588}$-YFP and myr-$HTT_{553\text{-}588}$-YFP could not be detected when the essential N-terminal glycine was substituted to an alanine (G553A) or serine (G553S). Non-myr-$HTT_{553\text{-}588}$-YFP migrates a higher molecular weight than myr-$HTT_{553\text{-}588}$-YFP. Cleavage bands correlating to myristoylated and non-myristoylated HTT could not be detected when caspase cleavage was blocked by substituting D552 with glutamate. *- denotes unknown band.

Uncropped gel from Figure 3B

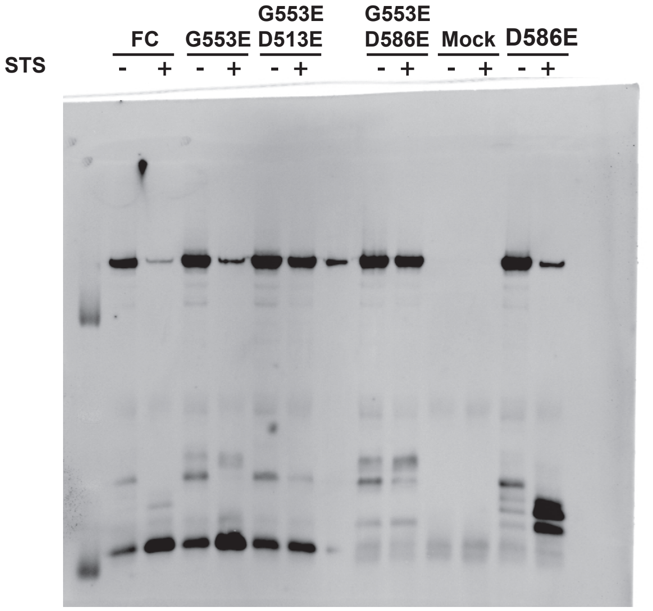

Uncropped gel from Figure 3D

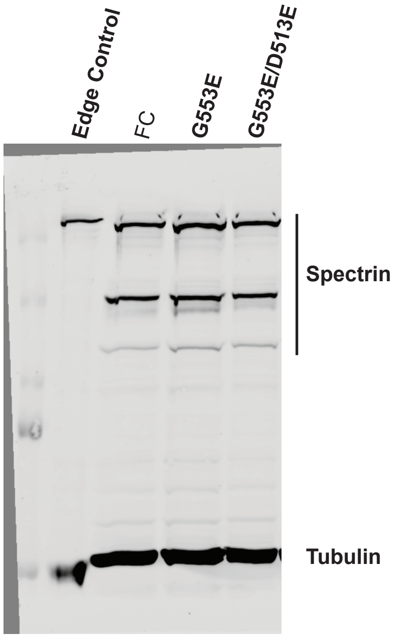

Supplemental References

1. Ehrnhoefer, D. E., Sutton, L. & Hayden, M. R. Small Changes, Big Impact: Posttranslational Modifications and Function of Huntingtin in Huntington Disease. *Neurosci.* **17,** 475–492 (2011).

2. Martin, D. D. O., Ladha, S., Ehrnhoefer, D. E. & Hayden, M. R. Autophagy in Huntington disease and huntingtin in autophagy. *Trends Neurosci.* **38,** 26–35 (2015).
